# Mesenchymal Stem Cells Expressing CES1 and Soluble TRAIL Activate CPT-11 and Induce Apoptosis in Lung Cancer Brain Metastatic Lesions

**DOI:** 10.1158/2767-9764.CRC-25-0209

**Published:** 2025-09-09

**Authors:** Dong Oh Kim, Eun Hwa Jang, Young Do Kwon, Ji Hye Yoo, Xiangyu Ma, Ki Hoon Kim, Dong Geun Hong, Chung Kwon Kim, Hyun Nam, Jung Won Choi, Geun-Hyoung Ha, Kyeung Min Joo

**Affiliations:** 1 Department of Anatomy and Cell Biology, Sungkyunkwan University School of Medicine, Suwon, South Korea.; 2 Medical Innovation Technology Inc. (MEDINNO Inc.), Seoul, South Korea.; 3 Department of Neurosurgery, Brain Tumor Center, Samsung Medical Center, Sungkyunkwan University School of Medicine, Seoul, South Korea.; 4 Stem Cell and Regenerative Medicine Center, Research Institute for Future Medicine, Samsung Medical Center, Seoul, South Korea.; 5 Department of Health Sciences and Technology, SAIHST, Sungkyunkwan University, Seoul, South Korea.; 6 Biomedical Institute for Convergence at SKKU (BICS), Sungkyunkwan University (SKKU), Suwon, South Korea.

## Abstract

We aimed to develop a novel therapeutic strategy for lung cancer brain metastases by leveraging the tumor-tropic properties of genetically engineered Wharton’s Jelly–derived mesenchymal stem cells (WJ-MSC) as vehicles for dual-agent gene therapy across the blood–brain barrier. WJ-MSCs were transiently engineered using lipid nanoparticle technology to coexpress soluble TRAIL (sTRAIL) and the prodrug-activating enzyme carboxylesterase 1 (CES1). *In vitro* analyses assessed transfection efficiency, therapeutic protein expression, apoptosis induction, and maintenance of stemness. Tumor-homing capacity was evaluated via EGFP labeling and intracerebral tracking. Therapeutic efficacy was tested in subcutaneous and intracerebral lung cancer xenograft models using bioluminescent imaging, histopathology, and IHC. *In vivo* treatment included intraperitoneal CPT-11 administration to assess synergy between CES1-mediated prodrug activation and sTRAIL-induced apoptosis. Modified WJ-MSCs exhibited preserved stem cell characteristics and strong tropism toward brain tumor sites. They secreted high levels of functional sTRAIL and CES1, enabling local activation of CPT-11 into SN-38 and inducing apoptosis through death receptor signaling (DR4/DR5). Combination therapy with WJ-MSCs-CES1.sTRAIL and CPT-11 significantly suppressed tumor growth in lung cancer brain metastasis models compared with control groups. The approach demonstrated selective cytotoxicity, minimal off-target effects, and favorable safety profiles. This study establishes a nonviral, transient gene delivery platform using autologous WJ-MSCs for dual-action gene therapy in lung cancer brain metastases. The combined use of CES1 and sTRAIL and enables precise tumor targeting and drug activation, offering a promising avenue for personalized, stem cell–based treatment strategies to improve outcomes in patients with brain metastatic lung cancer.

**Significance:**

This study presents a nonviral, stem cell–based therapy for brain metastatic non–small cell lung cancer using WJ-MSCs expressing sTRAIL and CES1. These engineered cells home to tumors, activate CPT-11, and induce apoptosis. The dual-action strategy significantly reduced brain tumor burden with minimal toxicity, demonstrating strong therapeutic potential.

## Introduction

Lung cancer remains a significant global health concern, particularly in Korea, where it is the leading cause of cancer-related deaths, and in the United States, where it ranks as the second highest cause (1, 2). According to reports from the NCI, lung and breast cancers were the most commonly diagnosed cancer types in the United States in 2020 (3). The International Agency for Research on Cancer states that lung cancer is the most prevalent cancer worldwide. Approximately 10% of patients with lung cancer are at risk of developing brain metastases, with lung cancer being the leading cause, accounting for 40% to 50% of cases, followed by breast cancer (15%–30%), melanoma (5%–20%), and colorectal cancer (3%–8%; ref. 4). Patients with lung cancer often exhibit symptoms such as memory and speech problems, seizures, and headaches, which contribute to a poor prognosis. Although approximately 80% of patients survive 5 years after diagnosis, the average survival time is less than 6 months (4). Lung cancer frequently leads to brain metastases, with an incidence rate of approximately 50% among patients with lung cancer, breast cancer, and melanoma (5). In non–small cell lung cancer (NSCLC), the incidence of brain metastases is particularly high, with an average survival time of approximately 15 months after diagnosis (6). Symptoms of lung cancer brain metastases vary and may include headaches, nausea, seizures, speech and sensory disturbances, paralysis, muscle weakness, memory impairment, and reduced concentration (7). Owing to this clinical complexity, research into treatments for lung cancer brain metastases has been actively pursued, focusing mainly on symptomatic management and tumor-directed therapies (8). Symptomatic management often involves the use of corticosteroids (9), which can cause side effects such as edema, hypertension, unstable blood glucose levels, and decreased bone density (9). However, tumor-directed therapies include surgical resection, radiotherapy, immunotherapy, targeted therapy, and chemotherapy, each of which presents diverse side effects for patients (10). Mesenchymal stem cells (MSC) have gained attention in the fields of tissue engineering and regenerative medicine owing to their multipotency and ability to differentiate into various cell types, including chondrocytes, adipocytes, myofibroblasts, osteoblasts, and neurons (11). One of the promising sources of MSCs is human Wharton’s jelly (WJ-MSC), which can be easily harvested without ethical concerns and exhibits immunoprivileged properties and high differentiation potential (12, 13). Additionally, MSCs possess tumor tropism, making them ideal candidates for targeted drug delivery (14). Studies have demonstrated that MSCs are effective drug delivery vehicles, particularly in cases of malignant glioma (15). This property has spurred extensive research, especially in cardiovascular and neurologic diseases, owing to MSCs’ low immunogenicity, versatile differentiation capabilities, and ability to migrate to tumor sites (16). In the treatment of brain metastases, chemotherapy is often limited by the blood–brain barrier, which prevents therapeutic agents from reaching the brain, reducing treatment efficacy and leading to low systemic response rates (17). Thus, more effective therapies for malignant cancers are urgently needed. Gene therapy has emerged as a promising approach, focusing on apoptosis-related genes that selectively target cancer cells without damaging normal cells. Carboxylesterase is an enzyme approved for clinical use that can convert the prodrug CPT-11 (irinotecan) into its active form, SN-38 (7-ethyl-10-hydroxy-camptothecin; ref. 18). Moreover, TNF-related apoptosis-inducing ligand (TRAIL) selectively induces apoptosis in cancer cells by binding to death receptors DR4 and DR5 (19). This process involves the recruitment of the adapter protein Fas-associated death domain protein and the formation of the death-inducing signaling complex (DISK), which leads to the activation of initiator caspase-8 and subsequent downstream apoptotic pathways (20). To enhance the therapeutic efficacy of TRAIL, we used a secreted form of TRAIL (sTRAIL), which includes a ligand for the Flt3 tyrosine kinase receptor, facilitating the secretion of various proteins (21). In this study, we used lipid nanoparticles (LNP) to genetically modify WJ-MSCs to express carboxylesterase 1 (CES1), sTRAIL, and CES1.sTRAIL. We evaluated the therapeutic efficacy of these modified WJ-MSCs in conjunction with CPT-11 through various injection methods and investigated the homing system of WJ-MSCs.

## Materials and Methods

### Cell culture

NSCLC cell lines (H460. PC14PE6) were obtained from Sungkyunkwan University and cultured in RPMI 1640 medium (Thermo Fisher Scientific) supplemented with 10% FBS (Gibco) and 1% penicillin/streptomycin (Corning). The cells were maintained in a humidified incubator at 37°C with a controlled CO_2_ atmosphere. Subculturing was performed every 3 to 4 days to ensure the continuous growth and viability of lung cancer cells. Plasmid DNA was transfected into NSCLC cells using pLenti CMV Puro LUC (Addgene, plasmid #17477), followed by selection using puromycin. WJ-MSCs were cultured in minimum essential medium alpha supplemented with 10% FBS and 0.1% gentamicin. The cells were incubated at 37°C in a CO_2_-controlled environment. Subculturing was conducted at 3 to 4 days intervals to maintain the growth and vitality of the WJ-MSCs.

### LNP-mRNA formulation and transfection

To deliver CES1, sTRAIL, and enhanced GFP (EGFP) mRNAs, LNPs were formulated using a microfluidic mixing platform (NanoGenerator Flex-S System, Precision NanoSystems), as previously described (22). Lipids (ionizable lipid, 1,2-distearoyl-sn-glycero-3-phosphocholine, cholesterol, and polyethylene glycol-lipid) in ethanol were mixed with mRNA in 25 mmol/L citrate buffer (pH 4.0) at a 3:1 aqueous:ethanol flow rate, enabling the self-assembly of mRNA-LNPs. EGFP mRNA (CleanCap EGFP mRNA, cat. no. L-7601) was purchased from TriLink Biotechnologies. CES1 and sTRAIL mRNAs were synthesized by TriLink Biotechnologies. Particle size and polydispersity index were measured using a Zetasizer Nano ZS (Malvern Instruments), and encapsulation efficiency was determined using RiboGreen RNA Assay Kit (Thermo Fisher Scientific) and a SpectraMax iD5 plate reader (Molecular Devices). For *in vitro* transfection, H460-Luc cells (3 × 10^5^) and WJ-MSCs (4 × 10^5^) were seeded into 6-well plates in 2 mL of complete growth medium and incubated overnight at 37°C with 5% CO_2_. The following day, the medium was replaced, and preformulated LNPs containing CES1, sTRAIL, or EGFP mRNA were added at a dose of 250 ng per well. After 6 hours of incubation, the cells were washed twice with PBS, the medium was replaced with fresh medium, and the cells were harvested for further analyses.

### CES1 measurement of CES1 enzymatic activity

CES1 activity was measured using a fluorometric Carboxylesterase Activity Assay Kit (Abcam, ab273314) according to the manufacturer’s instructions. Briefly, 4 × 10^5^ cells or 10 mg of tissue were lysed in 100 μL of CE Assay Buffer on ice for 10 minutes. The lysates were centrifuged at 10,000 × *g* for 15 minutes at 4°C to remove debris, and the supernatants were collected. Protein concentrations were determined using the Bradford assay. A standard curve was generated using serial dilutions of the CE standard. Samples and standards (50 μL/well) were loaded in duplicate into black 96-well plates, followed by the addition of 50 μL of CE Reaction Mix to each well. The reaction was monitored kinetically using a fluorescence microplate reader (Infinite M200, Tecan), and CES1 activity was calculated from the standard curve and expressed as pmol/minutes/mL.

### Western blotting

Western blotting samples were detached using 0.25% trypsin-EDTA (Thermo Fisher Scientific). Cell lysis was performed using RIPA buffer (Invitrogen) with a cell lysis process involving tapping every 10 minutes for a total of three times. Subsequently, centrifugation was performed under the following conditions: RPM, 13,000 rpm; temperature, 4°C; and time, 20 minutes. After cell lysis, supernatants were collected in Eppendorf tubes. Quantitative analysis was performed using the Bradford assay (Bio-Rad). Electrophoresis, protein transfer, and antibody detection were performed as follows. Western blotting samples were prepared by diluting the protein in 4× sample buffer to a final concentration of 1× using distilled water. A total of 30 μg of protein was loaded per well, and after the electrophoresis step, protein transfer was conducted for 90 minutes. Proteins were detected using antibodies against Flt-3L (Santa Cruz Biotechnology; cat. no. sc-365565), CES1 (Abcam; cat. no. ab45957), EGFP (Santa Cruz Biotechnology; cat. no. sc-9996), and β-actin (Cell Signaling Technology; cat. no. 4967), all at a 1:1,000 dilution in 1× TBST containing 5% BSA. The secondary antibodies used were anti-mouse IgG (GenDEPOT, cat. no. SA001-500) and anti-rabbit IgG (CES1; GenDEPOT, cat. no. SA002-500) diluted to 1:10,000. Protein detection was performed using the ECL Prime Western Blotting System, and the results were visualized using an X-ray film (Agfa).

### Experimental animals for lung cancer brain metastasis mouse modeling

Animal experimental procedures were approved by the Institutional Animal Care and Use Committee of the Laboratory Animal Research Center at the Sungkyunkwan University School of Medicine (SKKUIACUC2022-05-26-1). Before *in vivo* injection, H460 and PC14PE6-Luc cells were seeded at 4 × 10^5^ cells/well in a 12-well plate. The following day, luciferin was added to each well at a concentration of 5 mmol/L, and luminescence was confirmed using IVIS imaging to verify cell expression in both cell lines. Experiments were conducted using male BALB/c nude mice at 6 weeks of age. Initially, intracerebral (IC) injections were performed on the right hemisphere of the mice using H460- (1 × 10^5^ cells in μL HBSS) and PC14PE6-Luc (4 × 10^5^ cells in μL HBSS) cells. A body weight decrement of 20% or more was defined as severe suffering, serving as a potential parameter for humane endpoint decisions.

### Therapeutic efficacy of WJ-MSCs with therapeutic genes *in vivo*

Seven days after the H460-Luc cell (1 × 10^6^ cells in 100 μL of HBSS) implantation on the right flank, the mice were randomized into five groups and treated with WJ-MSCs + PBS, WJ-MSCs + CPT-11, WJ-MSCs-CES1 + CPT-11, WJ-MSCs-sTRAIL + CPT-11, and WJ-MSCs-CES1.sTRAIL + CPT-11. The mice, divided into groups, received intratumoral injections of WJ-MSCs (5 × 10^5^ in 100 μL of HBSS). On the days following injection, the mice received intraperitoneal injections of either PBS or CPT-11 (15 mg/kg) daily for 5 days. To measure tumor volume, the mice were euthanized 35 days after tumor cell insertion.

### MSC migration assay *in vivo*

BALB/c nude mice were stereotaxically implanted with H460-Luc (1 × 10^5^ cells in 5 μL of HBSS) in the white matter of the right hemispheres (approximately ML + 2.0 mm, AP + 0.5 mm, DV −3.0 mm from the bregma). Seven days after tumor/PBS implantation, all mice were administered WJ-MSCs-EGFP (5 × 10^5^ cells in 5 μL of HBSS) into the left lateral ventricle (approximately ML -1.0 mm, −AP−-0.0 mm, DV −3.0 mm from the bregma). Finally, the incision was sutured using 6-0 black silk sutures. After intracerebroventricular (ICV) injection of WJ-MSCs-EGFP spheres, two mice per group were sacrificed at 0, 6, 12, 24, and 48 hours. Subsequently, the results were confirmed through IHC fluorescent staining of brain tissue.

### Evaluation of the therapeutic efficacy of CES1 and sTRAIL mRNAs in brain metastasis of lung cancer

Six-week-old male BALB/c nude mice were distributed into four groups and treated as follows: Mice were randomly assigned to five groups and received IC injections in the right hemisphere. Each group was further subdivided into H460 + PBS, H460 + CPT-11, H460-CES1 + CPT-11, H460-sTRAIL + CPT-11, and H460-CES1.sTRAIL + CPT-11 and received 1 × 10^5^ cells in 5 μL of HBSS. H460-Luc cells were transfected with the LNP-mRNA. Following cell transplantation, CPT-11 (15 mg/kg) was administered via intraperitoneal injection for five consecutive days.

### Therapeutic efficacy studies of WJ-MSCs with modified genes in a lung cancer brain metastasis model *in vivo*

H460-Luc (1 × 10^5^ cells in 5 μL of HBSS) cells were stereotaxically implanted in the white matter of the right hemisphere of the brains of athymic nude mice (approximately ML + 2.0 mm, AP + 0.5 mm, DV −3.0 mm from the bregma). After 7 days, the mice in the WJ-MSCs + PBS, WJ-MSCs + CPT-11, WJ-MSCs-CES1 + CPT-11, WJ-MSCs-sTRAIL + CPT-11, and WJ-MSCs-CES1.sTRAIL + CPT-11 groups received 5 × 10^5^ cells in 5 μL of HBBS given into the left lateral ventricle (approximately ML −1.0 mm, −AP -0.0 mm, DV −3.0 mm from the bregma). Following the injections, the mice were intraperitoneally administered either PBS or CPT-11 (15 mg/kg) daily for five consecutive days.

### Bioluminescence IVIS imaging

Cellular bioluminescence was measured by adding 5 mmol/L luciferin in PBS to cell culture plates. After a 10-minute incubation, bioluminescence readings were recorded. Bioluminescence of the tumor volume was measured as previously reported. Briefly, the mice received intraperitoneal injections of 5 mmol/L luciferin in PBS (Promega, P1043). After waiting for 20 minutes, the IVIS Optical Imaging apparatus was used to measure photon emission.

### IHC

The tissues underwent paraffin embedding and were sectioned into 4-μm sections. Following deparaffinization and dehydration, sections were subjected to microwave-based antigen retrieval for 20 minutes. Endogenous peroxidase activity was quenched using a 3% hydrogen peroxide solution. Subsequently, the tissue samples were blocked with a mixture of 5% BSA and 2% normal goat serum for 1 hour. After completing the blocking step, we used an avidin–biotin blocking kit (Vector, SP-2001) in accordance with the manufacturer’s instructions. The kit was appropriately diluted in the same blocking solution and incubated at room temperature for 15 minutes to neutralize the endogenous avidin and biotin present in the tissue. The primary antibodies used were Ki67 (Abcam; cat. no. ab16667; 1:250) and cleaved caspase-3 (Cell Signaling Technology; cat. no. 9661; 1:400). The secondary antibody used was goat anti-rabbit IgG H&L (horseradish peroxidase; Abcam; cat. no. ab205718; 1:500). Slides were visualized using 3,3′-diaminobenzidine staining, and hematoxylin was used for nuclear staining. The ratio of 3,3′-diaminobenzidine-positive cells to hematoxylin-stained cells was determined using color deconvolution with the ImageJ image analysis software (NIH). In the initial step, a mixture of primary antibodies, specifically Ki67 (Abcam; cat. no. ab16667; 1:250), cleaved caspase-3 (Cell Signaling Technology; cat. no. 9661; 1:400), CD31 (Abcam; cat. no. ab28365; 1:2,000), and EGFP (Santa Cruz Biotechnology; cat. no. sc-9996; 1:500). The secondary antibodies used were Alexa Fluor 594–conjugated anti-rabbit IgG (Invitrogen; cat. no. VA295503; 1:200) and Alexa Fluor 488–conjugated anti-mouse IgG (Invitrogen; cat. no. A28175; 1:300), which were incubated at room temperature for 1 hour.

### Statistical analysis

The data are reported as the mean ± S.E.M., and statistical significance was determined using one-way or two-way ANOVA, denoted as *, *P* < 0.05; **, *P* < 0.001; and ***, *P* < 0.0001 (GraphPad Prism v9.0).

### Data availability

The data generated in this study are available upon request from the corresponding author.

## Results

### *In vitro* synthesis and transfection efficiency of CES1 and sTRAIL mRNAs in WJ-MSCs

To evaluate the efficacy of the mRNA delivery systems, GFP mRNA was transfected into WJ-MSCs using three methods: Lipofectamine, JetMESSENGER, and LNPs. Transfection efficiency was assessed by fluorescence microscopy and flow cytometry, which revealed that LNPs achieved substantially higher delivery efficiency, with GFP-positive cell rates increasing by approximately 10% to 70% compared with Lipofectamine and JetMESSENGER (Supplementary Fig. S1A and S1B). To confirm the successful synthesis and delivery of therapeutic mRNAs, *in vitro*–transcribed CES1 and sTRAIL mRNAs were analyzed by agarose gel electrophoresis to verify their integrity and size (Supplementary Fig. S1C). Western blot analysis demonstrated the robust protein expression of CES1 and sTRAIL (FLT-3L) in WJ-MSCs transfected with LNP-encapsulated mRNAs (Supplementary Fig. S1D). CES1 enzymatic activity was significantly increased in transfected WJ-MSCs compared with the control group, indicating functional expression (Supplementary Fig. S1E). Additionally, ELISA confirmed the enhanced secretion of sTRAIL protein into the culture medium following LNP-mRNA transfection (Supplementary Fig. S1F). These results validated the superior efficiency of LNP-mediated mRNA delivery into WJ-MSCs and confirmed the successful expression and functionality of CES1 and sTRAIL mRNAs *in vitro*.

Furthermore, time-course analysis of CES1 and sTRAIL expression following LNP-mediated mRNA transfection revealed that both mRNAs induced sustained protein expression and functional activity in WJ-MSCs (Supplementary Fig. S2). CES1 protein levels and carboxylesterase activity increased progressively, peaking at 72 hours, and remained detectable for up to 120 hours. Similarly, the intracellular expression and secretion of the Flt-3L–tagged sTRAIL protein were robustly induced, with the highest levels observed at 72 hours and maintained through 120 hours. These findings further support the efficient and prolonged functional expression of therapeutic mRNAs delivered via LNPs to WJ-MSCs.

### Phenotypic integrity and multipotency of WJ-MSCs following LNP-CES1.sTRAIL mRNA transfection

To assess the phenotypic stability of WJ-MSCs after LNP-CES1.sTRAIL transfection, morphologic and functional analyses were performed. Phase-contrast microscopy showed no significant morphologic changes in WJ-MSCs between the naïve and transfected groups at 0, 6, 24, and 48 hours after transfection (Supplementary Fig. S3A). Flow cytometric analysis of surface antigen expression revealed that both groups retained typical MSC marker profiles: positive for CD44, CD73, CD90, CD105, and CD166 and negative for hematopoietic and immune markers, including CD11b, CD14, CD19, CD34, CD45, and HLA–DR isotype (Supplementary Fig. S3B). Transfection with CES1 and sTRAIL mRNAs did not alter surface marker expression compared with naïve WJ-MSCs. Furthermore, multipotency was preserved in the transfected cells, as evidenced by successful adipogenic, osteogenic, and chondrogenic differentiation after 21 days of lineage-specific induction. Oil Red O, Alizarin Red, and Alcian Blue staining confirmed the presence of lipid droplets, calcium deposits, and sulfated proteoglycans, respectively (Supplementary Fig. S3C). These findings indicated that transfection with CES1 and sTRAIL mRNAs did not compromise the stemness or differentiation capacity of WJ-MSCs.

### CES1 and sTRAIL demonstrated therapeutic efficacy against lung cancer *in vivo*

**Figure 1.**
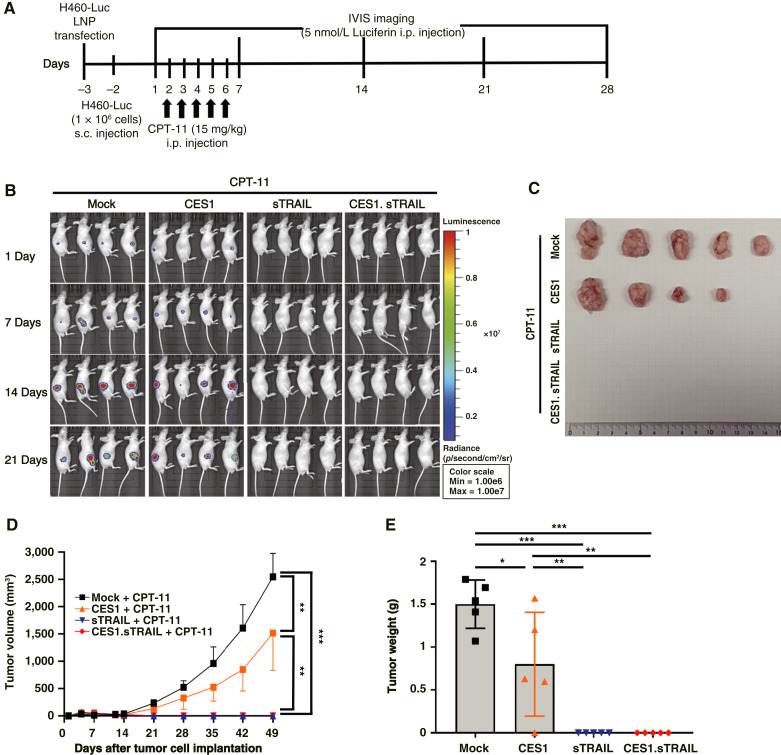
*In vivo* of therapeutic efficacy of mRNA-CES1 and sTRAIL for H460-Luc. **A,** Schematic illustrates an *in vivo* experiment. **B,** Bioluminescence imaging was performed using IVIS Optical Imaging equipment during the experiment. **C,** Representative subcutaneous tumors in the control and gene-transfected treatment groups. **D,** Graphs depicting the changes in tumor volume for each group. **E,** Graphs showing the changes in tumor weight for each group. All results are expressed as the mean ± SD; *N* = 4 for all groups. *P* values of statistical significance are represented as *, *P* < 0.05; **, *P* < 0.001; and ***, *P* < 0.000.

Building upon the successful validation of CES1 and sTRAIL efficacy in previous *in vitro* studies, we investigated their therapeutic potential in an *in vivo* setting through subcutaneous injections in BALB/c nude mice (Fig. 1A). H460-Luc cells were divided into CES1, sTRAIL, and combination groups and subjected to LNP-mRNA transfection. Each group of mice received subcutaneous injections in the right flank, followed by intraperitoneal injections of either CPT-11 (15 mg/kg) or PBS for 5 days during the experiment. IVIS imaging was conducted at 1-, 7-, 14-, and 21-day intervals following the intraperitoneal injection of 5 mmol/L luciferin (Fig. 1B). Over time, a significant decrease in tumor bioluminescence was observed in the sTRAIL and combination groups. After the experiments, the tumors were harvested (Fig. 1C). Unlike the control and CES1 groups, no visible tumors were detected in the sTRAIL and combination groups. This observation was further confirmed by measuring tumor size in each group (Fig. 1D). Tumor weights were significantly different between the control and CES1 groups, with more pronounced differences observed between the sTRAIL and combination groups (Fig. 1E). These findings demonstrated that the therapeutic efficacy of CES1, sTRAIL, and the combination group was clearly evident in an *in vivo* setting, highlighting their potential as promising candidates for lung cancer treatment.

Importantly, these *in vivo* results were consistent with our previous *in vitro* data (Supplementary Fig. S4), in which treatment with CES1 and sTRAIL mRNAs, particularly in combination with CPT-11, significantly reduced the viability of H460-Luc lung cancer cells. This concordance between *in vitro* and *in vivo* findings further supports the therapeutic potential of CES1 and sTRAIL as effective agents against lung cancer.

### WJ-MSCs with LNP-mRNA transfection demonstrate anticancer potential *in vivo*

**Figure 2.**
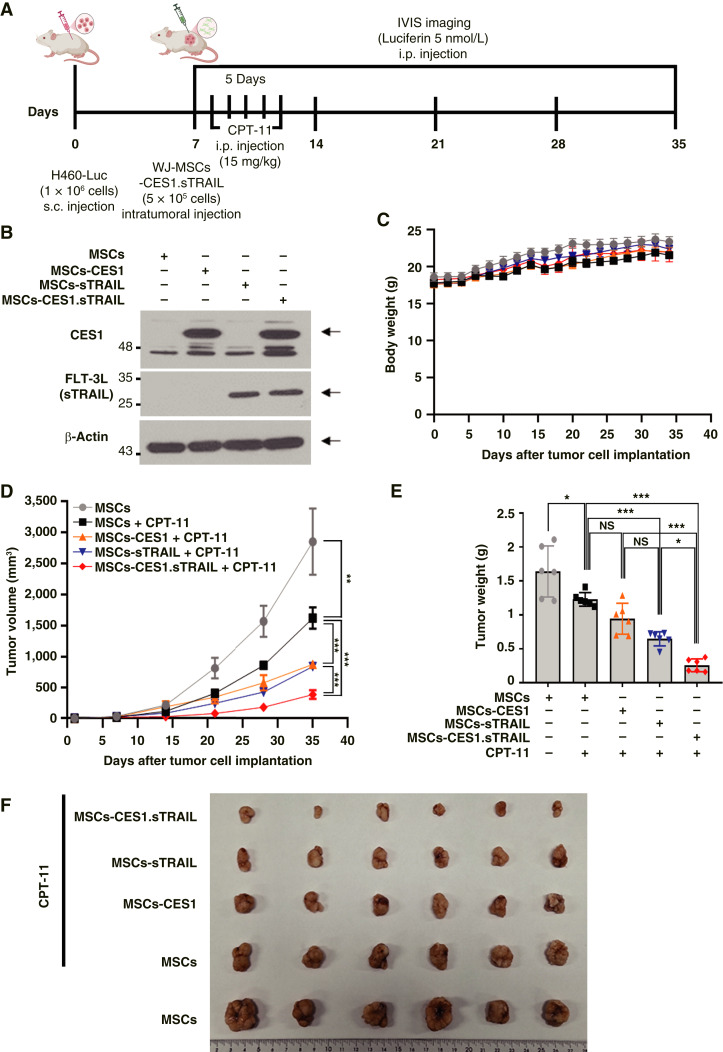
*In vivo* of therapeutic efficacy of LNP-mRNA-MSCs for H460-Luc. **A,** Schematic representation of an *in vivo* experiment. **B,** Confirming CES1 and FLT-3L expression in Western blot results of WJ-MSCs transfected with LNP-mRNA. **C,** A graph shows changes in the body weight of nude mice during WJ-MSCs-mRNA treatment. **D,** Average tumor volume. **E,** Average tumor weight. NS, not significant. **F,** Representative subcutaneous tumors in the control and treatment groups. All results are expressed as the mean ± SD; *N* = 6 for all groups. *P* values of statistical significance are represented as *, *P* < 0.05; **, *P* < 0.001; and ***, *P* < 0.0001.

After confirming the *in vivo* efficacy of CES1, sTRAIL, and their combination, we investigated the potential therapeutic effects of WJ-MSCs (Fig. 2A). First, WJ-MSCs underwent LNP-mediated mRNA transfection to create groups for mRNA-CES1, sTRAIL, and their combination. Western blot analysis (Fig. 2B) was performed to confirm the successful expression of the transfected genes in each group. Seven days after the subcutaneous injection of H460-Luc cells into the right flank of mice, intratumoral injections of WJ-MSCs from each group were administered. On the day after WJ-MSCs administration, intraperitoneal injections of CPT-11 were initiated. No significant changes in body weight were observed among the groups throughout the experiment, with all the groups showing a general increase in body weight (Fig. 2C). Further analysis revealed significant differences in tumor volume and weight among the groups (Fig. 2D and E). Compared with the control group, a substantial reduction in tumor size was observed in the group treated with CPT-11 alone, as well as in the CES1, sTRAIL, and combination groups. These findings confirmed that CPT-11 monotherapy led to a statistically significant decrease in tumor burden. At the conclusion of the experiment, tumors were harvested to observe variations in tumor size between the groups (Fig. 2F). These findings strongly suggest that *in vivo* therapeutic interventions using WJ-MSCs have significant potential.

### Establish a model of brain metastasis through IC injection of NSCLC

**Figure 3.**
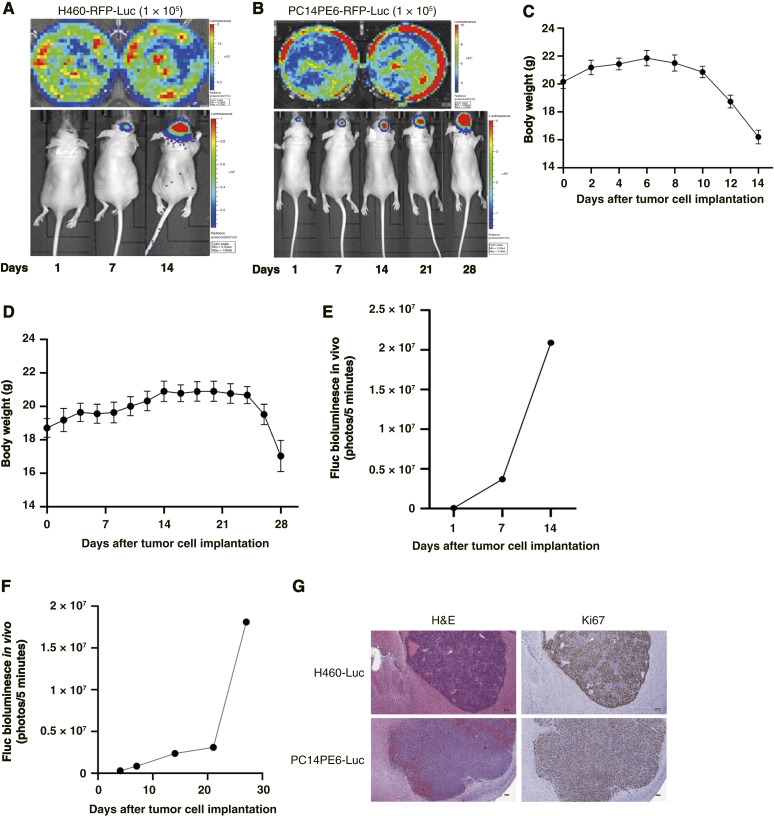
Modeling lung cancer brain metastasis using NSCLC. **A** and **B,** Tumor growth was monitored with luciferase expression via IVIS Spectrum imaging at different time points, and mice were imaged 10 minutes after luciferin intraperitoneal injection. **C** and **D,** Changes in mouse body weight after NSCLC injection. **E** and **F,** Plot of bioluminescence signal changes showing *in vivo* tumor growth. **G,** Histologic analysis of tumor tissues included hematoxylin and eosin (H&E) and Ki67 staining. Scale bar, 100 μm. All results are expressed as the mean ± SD.

We established a mouse brain model of NSCLC to investigate potential therapies for lung cancer with brain metastasis. We administered IC injections of H460-Luc and PC14PE6-Luc cells into the right hemisphere of BALB/c nude mice. Subsequently, IVIS imaging results were obtained after intraperitoneal injection of 5 mmol/L luciferin (Fig. 3A and B). The experiments were terminated if the body weight of any mouse in any group fell below 20%. When monitoring body weight changes, it was evident that each group had H460-Luc cells reaching this threshold at 14 days and PC14PE6-Luc cells at 28 days (Fig. 3C and D). Analysis of the luciferase expression levels for each group revealed increased luciferase activity at 7 and 20 days after injection (Fig. 3E and F). Following the conclusion of the experiments, we harvested mouse brain tissue and conducted staining to examine the areas of tumor pathology (Fig. 3G). Consequently, the experimental design was tailored to the expected survival period of each group.

### Evaluation of therapeutic efficacy following LNP-mRNA transfection after establishing a brain metastasis model

**Figure 4.**
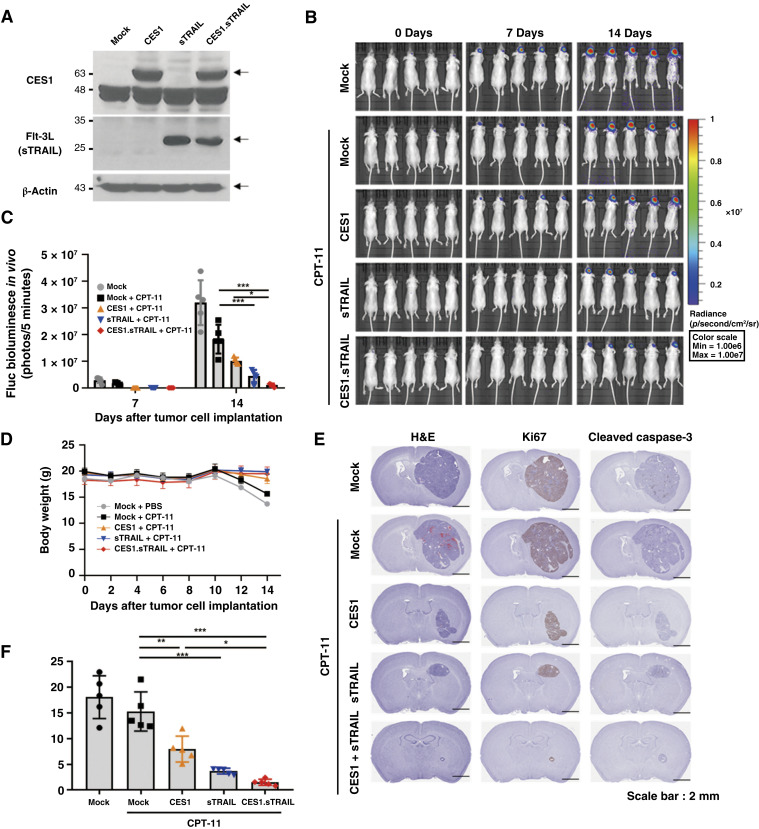
Therapeutic effects of treatment genes on lung cancer brain metastasis *in vivo*. **A,** Confirming CES1 and FLT-3L expression in Western blot results of H460-Luc cells transfected with LNP-mRNA. **B,** Bioluminescence imaging was conducted using IVIS Optical Imaging equipment during the experiment. The bioluminescent image of the first mouse in the mock day 7 condition is also presented for the day 7 condition in Fig. 3A, as the images represent the same experimental conditions. **C,** Bioluminescent signals were quantified using the IVIS imaging system, and results were obtained at 7 and 14 days after injecting H460-Luc cells for each group. **D,** Mouse body weight changes after injecting H460-Luc cells with transfection were assessed in each group of nude mice. **E,** Histologic analysis of tumor tissues in the H460-Luc model, stained with hematoxylin and eosin (H&E), Ki67, and cleaved caspase-3. **F,** A graph illustrating the area of cancer lesions in the mouse brain. All results are expressed as the mean ± SD; *N* = 5 for all groups. *P* values of statistical significance are represented as *, *P* < 0.05; **, *P* < 0.001; and ***, *P* < 0.0001.

After examining survival outcomes specific to different cell types, we assessed the therapeutic efficacy of mRNA-CES1, sTRAIL, and their combination in a lung cancer brain metastasis model. Initially, we performed LNP transfection of H460-Luc cells and validated their transfection success by examining normal protein expression via Western blotting (Fig. 4A). All groups demonstrated the anticipated protein expression levels, prompting the division of mice into five groups designated for IC brain injections. On the next day, intraperitoneal administration of CPT-11 (15 mg/kg) was initiated. IVIS imaging sessions were conducted at 0-, 7-, and 14-day intervals, with mice receiving intraperitoneal injections of 5 mmol/L luciferin (Fig. 4B). Our findings on day 7 indicated substantial variations in the fluorescence expression levels between the control group and the group receiving CPT-11 monotherapy (Fig. 4C). Furthermore, significant changes in body weight were observed during the course of the experiment. A reduction in body weight was observed after 10 days in the control and CPT-11 groups, whereas the remaining three groups did not show such reductions (Fig. 4D).

At the conclusion of our experiments, mouse brains from each group were harvested, and subsequent hematoxylin and eosin and IHC staining procedures were conducted to facilitate a comprehensive analysis (Fig. 4E). Visual inspection revealed reduced pathologic areas in the treatment groups compared with those in the control group. This reduction exceeded 15% in the control and combination groups (Fig. 4F). Consequently, our results underscore the apparent therapeutic efficacy of mRNA-CES1 and sTRAIL, and their combination in a brain metastasis model, signifying their promising potential for further exploration in cancer treatment.

### *In vivo* evaluation of the tumor-homing ability of WJ-MSCs

**Figure 5.**
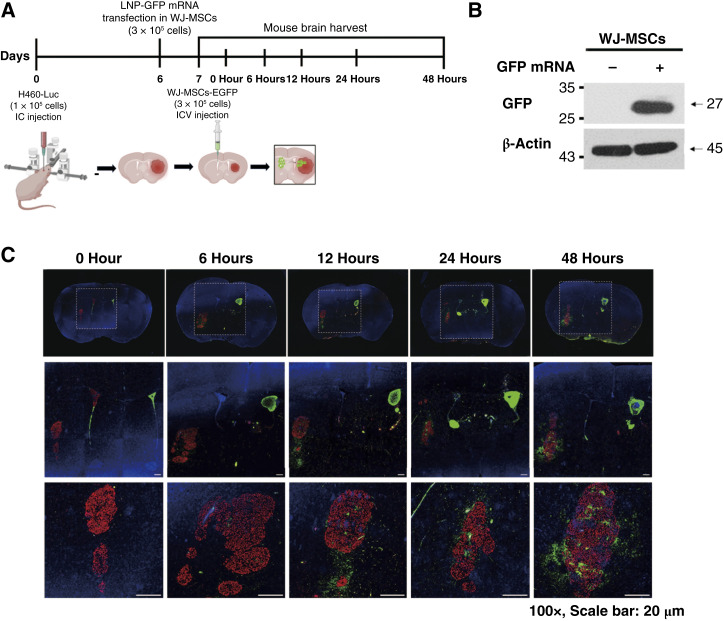
Therapeutic tumor-homing ability of MSCs. **A,** Schematic representation of an *in vivo* experiment. **B,** Confirming EGFP expression in Western blot results of WJ-MSCs transfected with LNP-mRNA. **C,** The CLSM results depict brain cross-sections from 0 to 48 hours after ICV injection of WJ-MSCs-mRNA-EGFP into mouse brains. (Green represents Alexa Fluor 488 fluorescence, and blue represents DAPI staining). CLSM, confocal laser scanning microscope; DAPI, 4′,6-diamidino-2-phenylindole, dihydrochloride.

To use the WJ-MSCs as a vehicle for cancer therapy, experiments were conducted to verify their homing capabilities. The experiment involved the administration of lung cancer cells in the right hemisphere of BALB/c nude mice, followed by intraventricular injection of WJ-MSCs in the opposite hemisphere. The time intervals were 0, 6, 12, 24, and 48 hours, during which the brains were harvested to examine the migration of WJ-MSCs (Fig. 5A). Prior to the administration of WJ-MSCs, we transfected the cells with LNP-mRNA-EGFP, and the degree of expression was confirmed through Western blot analysis (Fig. 5B). After ICV injection, we collected the mouse brains at various time points and assessed the results using IHC double staining (Fig. 5C). Over the 0 to 48-hour timeframe, we observed the migration of WJ-MSCs-EGFP (in green) toward the tumor (in red). Our findings confirm that WJ-MSCs can effectively serve as a vehicle for the delivery of treatment genes, further emphasizing their therapeutic potential.

### Therapeutic efficacy of LNP-mRNA–transfected WJ-MSCs in the treatment of lung cancer brain metastases *in vivo*

Based on the *in vitro* therapeutic efficacy of WJ-MSCs transfected with LNP-CES1 and LNP-sTRAIL mRNA against NSCLC cells (Supplementary Fig. S5), we evaluated the combined anticancer effects in two distinct NSCLC cell lines, H460-Luc and PC14PE6-Luc. Conditioned WJ-MSCs expressing CES1 and/or sTRAIL were cocultured with luciferase-labeled cancer cells in a transwell system, allowing the assessment of paracrine-mediated cytotoxicity without direct cell-to-cell contact. Upon treatment with CPT-11, a clinically used chemotherapeutic agent, both H460-Luc and PC14PE6-Luc cells exhibited a significant decrease in viability, with the greatest reduction observed in the cotreatment group, which received both CES1.sTRAIL mRNA-expressing WJ-MSCs and CPT-11. These findings highlight the synergistic potential of combining genetically engineered WJ-MSC therapy with chemotherapy to effectively suppress NSCLC cell growth *in vitro* and provide a strong rationale for further *in vivo* investigation.

**Figure 6.**
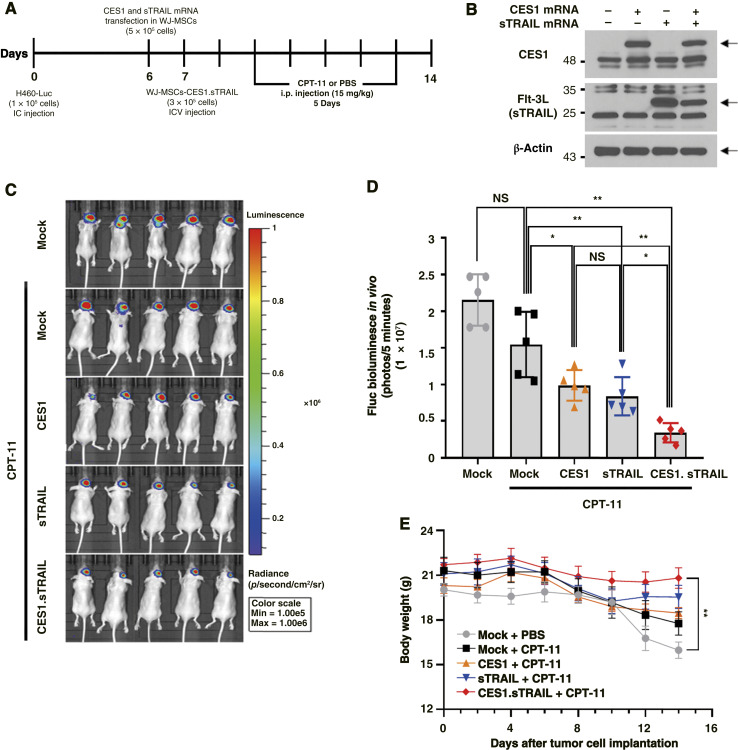
Therapeutic efficacy of CES1 and sTRAIL mRNAs delivered via WJ-MSCs in a lung cancer brain metastasis model. **A,** Schematic overview of the *in vivo* experimental design. **B,** Western blot analysis of CES1 and FLT-3L expression in WJ-MSCs transfected with LNP-encapsulated mRNAs. **C,** Bioluminescence imaging of tumor-bearing mice performed using the IVIS imaging system. **D,** Quantification of bioluminescent signals at 14 days after H460-Luc cell injection. NS, not significant. **E,** Body weight monitoring of nude mice throughout the treatment period. Data are presented as the mean ± SD; *n* = 5 per group. *P* values of statistical significance are represented as *, *P* < 0.05; **, *P* < 0.001.

Having validated the homing capabilities of WJ-MSCs, we evaluated the therapeutic potential of gene delivery using WJ-MSCs in treating lung cancer brain metastasis *in vivo* (Fig. 6A). Before therapeutic intervention, WJ-MSCs were transfected with LNP-mRNA, and the expression levels were verified to be normal through Western blotting (Fig. 6B). Following IC injection of H460-Luc cells into BALB/c nude mice, ICV injections of WJ-MSCs transfected with LNP-mRNA were administered to the opposite ventricle 7 days later. Subsequently, CPT-11 (15 mg/kg) was administered via intraperitoneal injection starting 2 days after WJ-MSCs administration. At day 14, we assessed the tumor fluorescence expression among the groups using IVIS imaging (Fig. 6C). The results indicated a significant difference in fluorescence expression between the control and combination groups, as illustrated in the graph (Fig. 6D). Subsequent observation of body weight changes in each group revealed a decreasing trend in all groups after 5 days. A significant difference was observed between the control and combination treatment groups (Fig. 6E).

**Figure 7.**
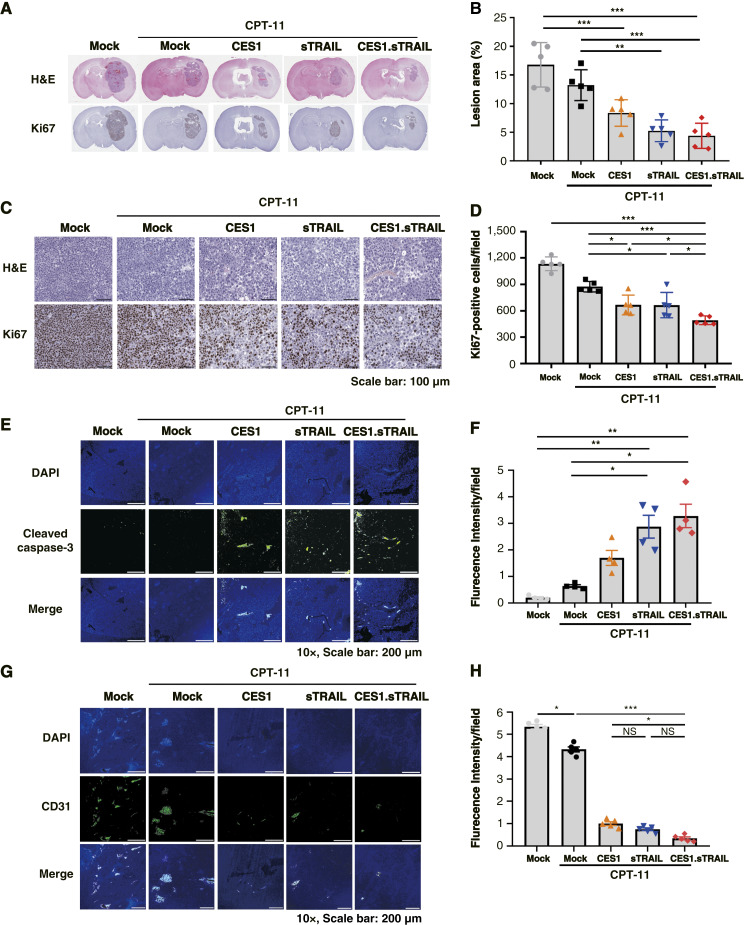
Histologic and molecular analyses of therapeutic outcomes in the brain metastasis model. **A,** Histologic examination of brain tumor tissues stained with hematoxylin and eosin (H&E) and Ki67. **B,** Quantification of tumor lesion area in the brain. **C,** Representative images of Ki67 immunostaining in brain tumor lesions. **D,** Quantification of Ki67-positive cells per group. **E–H,** Immunofluorescence staining for cleaved caspase-3 and CD31 in brain tissues, visualized using the ScanScope AT system (Leica Biosystems). Immunofluorescence staining for cleaved caspase-3 and CD31 in brain tissues, visualized using the ScanScope AT system (Leica Biosystems). **E** and **F,** Representative images and quantification of cleaved caspase-3–positive cells. **G** and **H,** Representative images and quantification of CD31-positive cells. Data are presented as the mean ± SD; *n* = 5 per group. *P* values of statistical significance are represented as *, *P* < 0.05; **, *P* < 0.001; and ***, *P* < 0.0001. DAPI, 4′,6-diamidino-2-phenylindole, dihydrochloride.

After the conclusion of the experiment, the mouse brains were harvested, and hematoxylin and eosin and IHC staining were performed to scrutinize the pathologic areas within the tissues (Fig. 7A). In the results, a significant distinction in tumor size was observed between the control and treated groups; however, no significant difference emerged among the treated groups (Fig. 7B). Additionally, when comparing Ki67-positive cells in each group, there was a noticeable decrease in the number of positive cells in the WJ-MSCs-CES1.sTRAIL group compared with the control group. These numerical values indicate a significant difference, as evidenced by the graphical representation (Fig. 7C and D). Furthermore, we conducted immunofluorescence staining of the tissues using cleaved caspase-3 and CD31 markers. The increased expression of cleaved caspase-3 in the combination group suggested that tumor apoptosis was induced by the gene delivery (Fig. 7E and F). Additionally, examining CD 31 marker expression, which is indicative of vascular density in malignant tissues, revealed higher expression in the control group (Fig. 7G and H). In conclusion, the WJ-MSCs-CES1.sTRAIL exhibited efficacy in treating lung cancer brain metastasis, with WJ-MSCs proficiently delivering the intended therapeutic genes to the target site. This underscores the promising prospects for gene therapy using stem cells.

## Discussion

Lung cancer remains the leading cause of cancer-related deaths worldwide (23). Brain metastasis is a malignant complication of lung cancer that contributes to the morbidity and mortality of patients with NSCLC (24). Despite aggressive interventions involving chemotherapy, surgery, and radiotherapy, malignant brain metastases from lung cancer continue to have a poor prognosis (25, 26). Given this challenging scenario, it is imperative to develop effective treatments to extend the life of patients with NSCLC and brain metastases (27). In this study, we used WJ-MSCs as cellular vehicles to deliver CES1 and sTRAIL mRNAs using LNPs combined with CPT-11 to treat lung cancer and lung cancer brain metastases. We found that both the CES1 and sTRAIL groups demonstrated great antitumor efficacy in lung cancer and lung cancer brain metastasis models. The CES1.sTRAIL group seemed to exhibit better efficacy than the CES1 and sTRAIL groups. We demonstrated that administration of CPT-11 alone resulted in only a slight antitumor effect. However, a significant reduction in tumor size was observed when CES1 and sTRAIL mRNAs were combined in both lung cancer and lung cancer brain metastasis models. Additionally, we observed a reduction in tumor cell proliferation and angiogenesis, along with an increase in apoptosis, in a lung cancer brain metastasis model. CPT-11 has remarkable efficacy in tumors, such as NSCLC and colon cancer, which are poorly responsive to conventional chemotherapy (28). Given that the activity of CPT-11 is relatively low, we used CE to convert CPT-11 into its active metabolite, SN-38, exhibiting a 1,000 times greater potency (29). The clinical use of SN-38 is typically constrained by its poor solubility and instability (30). After delivery of CE mRNA, the antitumor effect of CPT-11 significantly improved. In clinical settings, many patients receiving CPT-11 experience side effects such as diarrhea and gastrointestinal mucositis (31). Therefore, it is necessary to combine other treatments, such as gene therapy, to reduce the dose of CPT-11. sTRAIL is a powerful candidate for gene therapy owing to its ability to selectively kill cancer cells without damaging normal cells (32). In our study, the sTRAIL group demonstrated significant antitumor effects. According to previous studies, H460 cell lines are sensitive to sTRAIL because of the high expression of DR4 and DR5 (33, 34). However, most primary cancer cells resist TRAIL monotherapy, and a single use of TRAIL results in insufficient clinical efficacy (35, 36). Combining chemotherapies such as CPT-11 may enhance the antitumor effect and sensitivity of TRAIL to target cancer stem cells (37). Kim and colleagues (36) have found that human neural stem cells encoding CES1 and sTRAIL genes showed a significant anticancer effect on lung cancer brain metastases in the presence of CPT-11. Adipose-derived stem cells overexpress TRAIL and CES1, and CPT-11 significantly inhibits tumor growth and enhances apoptosis (38). CPT-11 and TRAIL recombinant proteins combine to inhibit tumor growth in both TRAIL-sensitive and TRAIL-resistant colon tumors (39–41). Although combining sTRAIL and chemotherapy did not show obvious significance in the brain metastasis model, the combination group seemed to have a better antitumor effect than the individual treatments. These findings suggest that combining CPT-11 with CES1 and sTRAIL genes may be an effective strategy for treating lung cancer and lung cancer brain metastases, with potential applicability to other cancer types. CPT-11, a topoisomerase-1 inhibitor, causes cancer cell death by binding to Topo I to create a CPT–Topo I–DNA ternary complex that prevents DNA synthesis (42, 43). TRAIL mediates apoptotic pathways by interacting with DR4/DR5, activating caspase-8 and downstream effectors (44, 45). Moreover, TRAIL inhibits angiogenesis by inducing apoptosis in DR5-expressing tumor endothelial cells and vascular smooth muscle cells (46). Ray and colleagues (47) have found that the combination of TRAIL and CPT-11 induces apoptosis by regulating the S-phase checkpoint, which is controlled by the Chk2-Cdc25A and Chk1-Cdc25A pathways, as well as by inhibiting Cdk2-associated kinase activity. In our study, we found that WJ-MSCs overexpressing sTRAIL and CES1 mRNAs, in combination with CPT-11, inhibited angiogenesis and proliferation, while enhancing apoptosis. This may prevent DNA synthesis and activate apoptosis-related signaling pathways in both tumor and endothelial cells. We used LNPs to deliver TRAIL and CES1 mRNA into WJ-MSCs to prevent gene integration into the genome and facilitate the targeted delivery of therapeutic genes to tumors. MSCs have been extensively studied as gene delivery systems for cancer therapy because of their natural homing tendency to solid tumors, gene delivery by genetic engineering, and ability to internalize nanoparticles (48, 49). In clinical trials, recombinant TRAIL protein and TRAIL receptor agonist mAbs showed disappointing results owing to their short half-lives, poor pharmacokinetics, and resistance of the cancer cells (49, 50). Many gene therapy clinical trials have used viral vectors to transfer cDNA, but this approach may lead to immunogenicity, and insertional mutagenesis and has been associated with reports of fatalities following viral vector administration (51). The use of mRNA, with its inherent advantages over DNA therapy, can be achieved by *in vitro* transcription techniques; it is fast and transiently expressed in the cytoplasm without the risk of integration into the genome, introducing a new dimension to the landscape of gene therapy (52). We used LNPs because of their large payload capacity, stability, and biocompatibility to deliver mRNA into WJ-MSCs and found that WJ-MSCs could successfully deliver sTRAIL and CES1 mRNAs via LNPs without affecting the viability and homing ability of WJ-MSCs (52). Delivering mRNA to WJ-MSCs enhances the safety and targeting of gene therapy and provides a novel approach for gene delivery. In conclusion, we found that WJ-MSCs carrying CES1 and sTRAIL mRNAs with CPT-11 had significant antitumor effects in both lung cancer and lung cancer brain metastasis models by promoting apoptosis and inhibiting angiogenesis and tumor cell proliferation. Additionally, we used LNPs to deliver mRNA to WJ-MSCs to avoid gene integration into the genome, and the homing ability of WJ-MSCs increased the specificity of gene therapy. Our study provides a new therapeutic strategy for lung cancer with brain metastases and a new method for safe and specific gene therapy.

## Supplementary Material

Supplementary Data Supplementary Figure 1

Supplementary Data Supplementary Figure 2

Supplementary Data Supplementary Figure 3

Supplementary Data Supplementary Figure 4

Supplementary Data Supplementary Figure 5
