# Supplementary material for: Mesenchymal Stem Cells Expressing CES1 and Soluble TRAIL Activate CPT-11 and Induce Apoptosis in Lung Cancer Brain Metastatic Lesions
Source: Cancer Res Commun. 2025 Sep 9;5(9):1552–65. doi: 10.1158/2767-9764.CRC-25-0209 (PMC12417980; doi:10.1158/2767-9764.CRC-25-0209)
Supplement: Supplementary Data — Supplementary Figure 1 [file crc-25-0209_supplementary_data_suppsf1.docx]

**
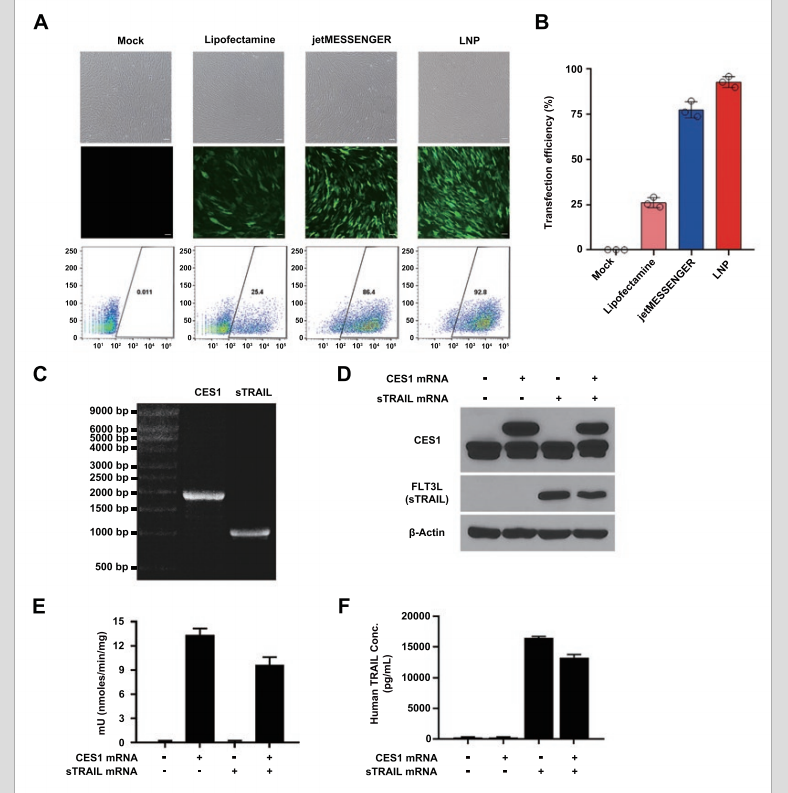
**

**Supplementary Figure 1. In vitro synthesis and transfection of CES1 and sTRAIL mRNAs into Wharton's jelly-derived mesenchymal stem cells (WJ-MSCs).**

**A,** Transfection efficiency of GFP mRNA into WJ-MSCs using three different delivery methods: Lipofectamine, JetMESSENGER, and LNP. Representative fluorescence microscopy images and flow cytometry (FACS) analysis are shown. **B,** Quantification of GFP-positive cells by flow cytometry following transfection using the indicated methods. **C**, Agarose gel electrophoresis of in vitro–transcribed CES1 and sTRAIL mRNAs. **D**, Western blot analysis of CES1 and sTRAIL (FLT-3L) protein expression in WJ-MSCs transfected with lipid nanoparticle (LNP)–encapsulated mRNAs. **E,** CES1 enzymatic activity following LNP–mRNA transfection in WJ-MSCs. **F**, Enzyme-linked immunosorbent assay (ELISA) of secreted sTRAIL levels after LNP–mRNA transfection in WJ-MSCs. Data are presented as mean ± standard deviation(SD); n = 3 per groups.
