# Supplementary material for: Mesenchymal Stem Cells Expressing CES1 and Soluble TRAIL Activate CPT-11 and Induce Apoptosis in Lung Cancer Brain Metastatic Lesions
Source: Cancer Res Commun. 2025 Sep 9;5(9):1552–65. doi: 10.1158/2767-9764.CRC-25-0209 (PMC12417980; doi:10.1158/2767-9764.CRC-25-0209)
Supplement: Supplementary Data — Supplementary Figure 2 [file crc-25-0209_supplementary_data_suppsf2.docx]

**
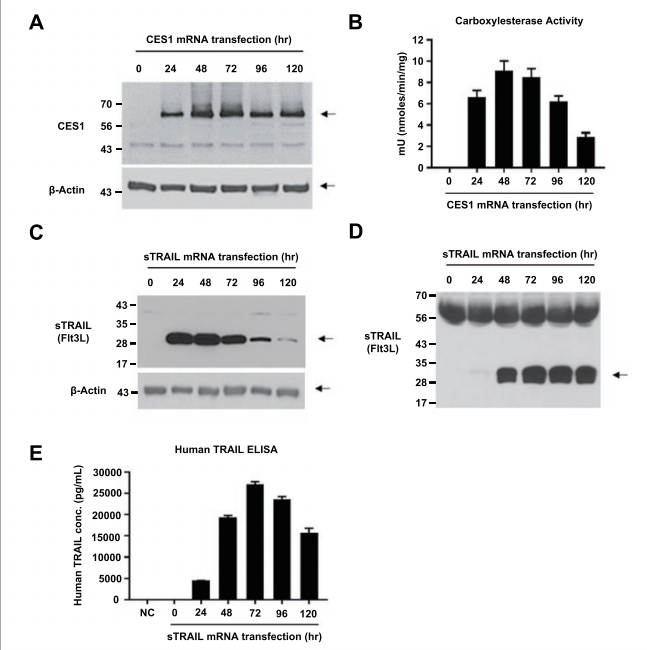
**

**Supplementary Figure 2. Expression and activity of CES1 and sTRAIL in WJ-MSCs after LNP-mRNA transfection.**

**A,** Western blot analysis of CES1 protein expression in WJ-MSCs at 0, 24, 48, 72, 96, and 120 hours following LNP-CES1 mRNA transfection. β-Actin was used as a loading control.
**B,** Carboxylesterase enzymatic activity in WJ-MSCs at indicated time points after LNP-CES1 mRNA transfection, measured by a CES1 activity assay. Data are presented as mean ± SD. **C,** Western blot analysis of sTRAIL (Flt3L-tagged) protein expression in WJ-MSCs at indicated time points after LNP-sTRAIL mRNA transfection. β-Actin was used as a loading control. **D,** Detection of secreted sTRAIL (Flt3L-tagged) in the culture supernatants by Western blot after LNP-sTRAIL mRNA transfection. (E) Quantification of human TRAIL concentration in the supernatants of WJ-MSCs transfected with LNP-sTRAIL mRNA by ELISA at the indicated time points. Data are presented as mean ± standard deviation(SD); n = 3 per groups.
