# Supplementary material for: Mesenchymal Stem Cells Expressing CES1 and Soluble TRAIL Activate CPT-11 and Induce Apoptosis in Lung Cancer Brain Metastatic Lesions
Source: Cancer Res Commun. 2025 Sep 9;5(9):1552–65. doi: 10.1158/2767-9764.CRC-25-0209 (PMC12417980; doi:10.1158/2767-9764.CRC-25-0209)
Supplement: Supplementary Data — Supplementary Figure 3 [file crc-25-0209_supplementary_data_suppsf3.docx]

**
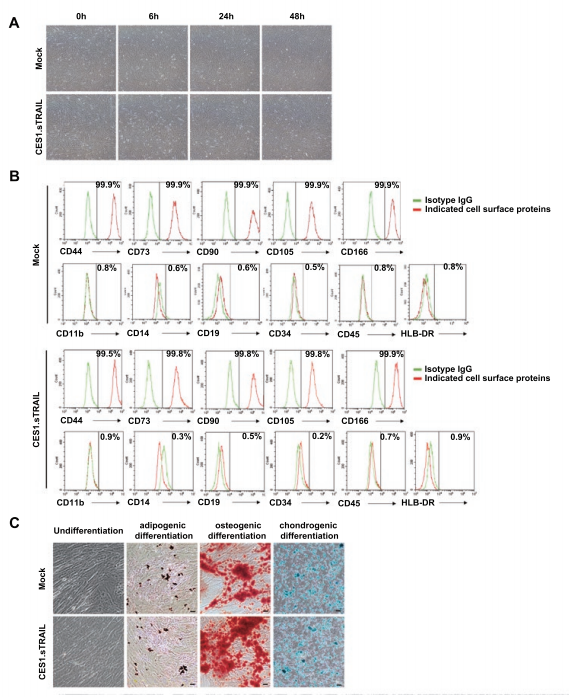
**

**Supplementary Figure 3. Characterization of LNP-CES1.sTRAIL Transfection in WJ-MSCs.**

**A,** After transfecting WJ-MSCs with naïve or LNP-CES1.sTRAIL, their morphology was observed at four different time points: 0, 6, 24 and 48 hours. **B,** After 48 hours of transfection, the expression of surface antigens in WJ-MSCs was analyzed using FACS. Green histograms indicate isotype controls, whereas red histograms represent signals for each specific marker. WJ-MSCs exhibited positive expression of MSC markers (CD44, CD73, CD90, CD105, and CD166) and negative expression of hematopoietic markers (CD11b, CD14, CD19, CD34, CD45, and HLA-DR), with no significant differences between the naïve and LNP-CES1.sTRAIL groups. **C**, Naïve and LNP-CES1.sTRAIL-transfected WJ-MSCs were cultured in adipogenic, osteogenic, and chondrogenic differentiation media for 21 days. Oil Red O and Alizarin Red staining confirmed lipid and calcium deposits, respectively, while Alcian Blue staining validated chondrogenic differentiation.
