# Supplementary material for: Mesenchymal Stem Cells Expressing CES1 and Soluble TRAIL Activate CPT-11 and Induce Apoptosis in Lung Cancer Brain Metastatic Lesions
Source: Cancer Res Commun. 2025 Sep 9;5(9):1552–65. doi: 10.1158/2767-9764.CRC-25-0209 (PMC12417980; doi:10.1158/2767-9764.CRC-25-0209)
Supplement: Supplementary Data — Supplementary Figure 4 [file crc-25-0209_supplementary_data_suppsf4.docx]

**
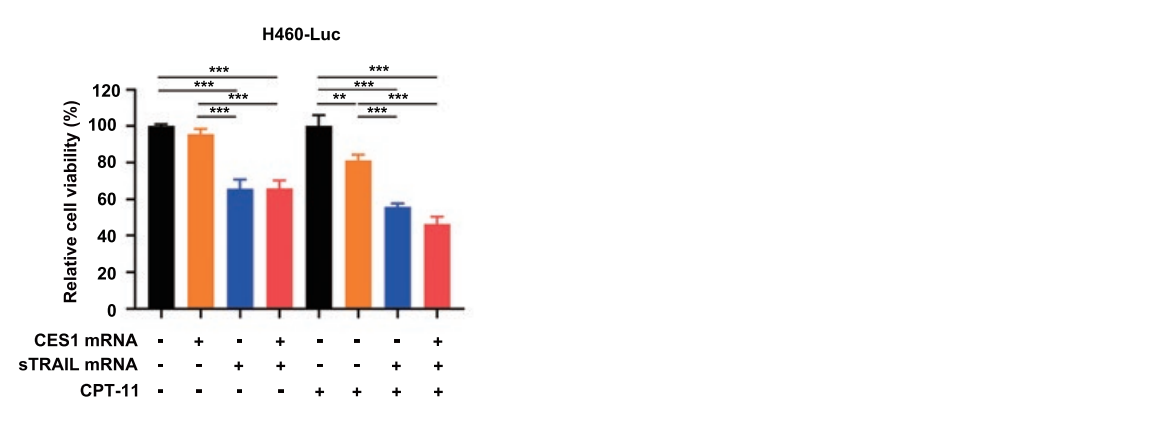
**

**Supplementary Figure 4. Synergistic cytotoxic effect of LNP-CES1.sTRAIL mRNA and CPT-11 in H460-Luc cells.**

H460-Luc cells were seeded in 6-well plates (5 × 10⁶ cells/well) and transfected with LNP-CES1 mRNA, LNP-sTRAIL mRNA, or LNP-CES1.sTRAIL mRNA (100 ng/well each). After 24 hours, transfected cells were reseeded into 24-well plates (6 × 10⁴ cells/well) and treated with or without CPT-11 (50 µM) for an additional 24 hours. Cell viability was assessed using an MTT assay. Data are presented as mean ± SD from triplicate wells. Expression of sTRAIL mRNA alone significantly reduced cell viability, and the combination of CES1.sTRAIL mRNA with CPT-11 resulted in the greatest reduction in viability, suggesting a synergistic therapeutic effect. Statistical analysis was performed using one-way ANOVA followed by Tukey’s post hoc test. *p < 0.05, **p < 0.01, ***p < 0.001.
