# Supplementary material for: Mesenchymal Stem Cells Expressing CES1 and Soluble TRAIL Activate CPT-11 and Induce Apoptosis in Lung Cancer Brain Metastatic Lesions
Source: Cancer Res Commun. 2025 Sep 9;5(9):1552–65. doi: 10.1158/2767-9764.CRC-25-0209 (PMC12417980; doi:10.1158/2767-9764.CRC-25-0209)
Supplement: Supplementary Data — Supplementary Figure 5 [file crc-25-0209_supplementary_data_suppsf5.docx]

**
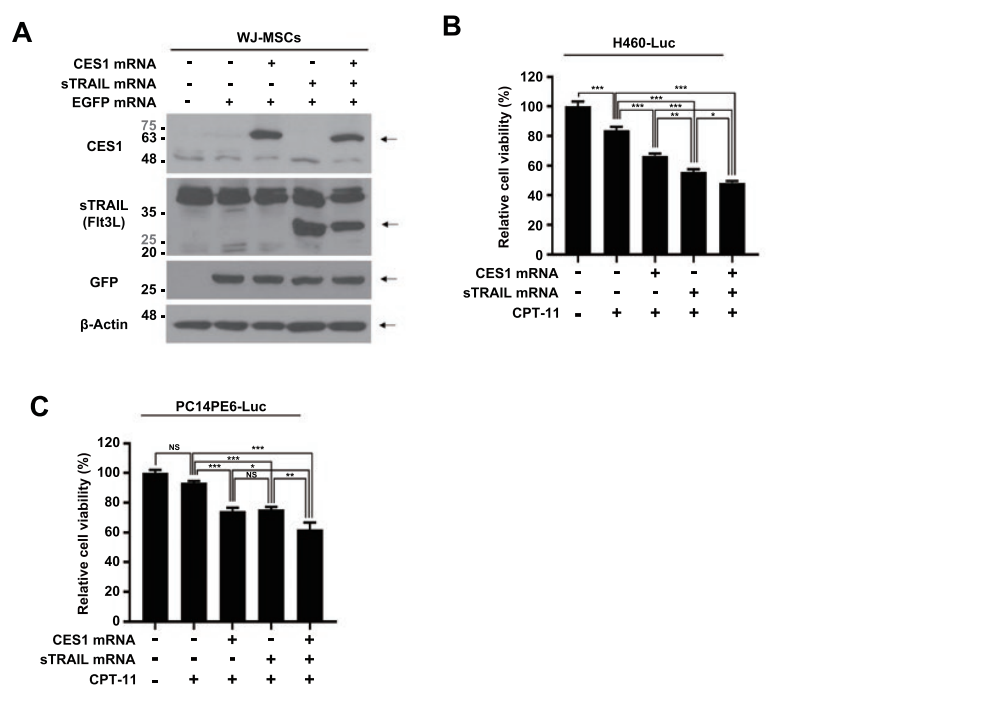
**

**Supplementary Figure 5. *In vitro* therapeutic efficacy of WJ-MSCs transfected with LNP-CES1 and LNP-sTRAIL mRNA against non-small cell lung cancer (NSCLC).**

**A,** Wharton’s jelly-derived mesenchymal stem cells (WJ-MSCs) were transfected with LNP-CES1 mRNA, LNP-sTRAIL mRNA, or both (100 ng/well each), and protein expression of CES1, sTRAIL, and GFP was assessed by Western blot analysis. β-Actin was used as a loading control. **B and C,** Conditioned WJ-MSCs were co-cultured with either H460-Luc (B) or PC14PE6-Luc (C) NSCLC cells in a Transwell system. After 24 hours of co-culture, cancer cells were treated with or without CPT-11 (50 μM), and relative cell viability was assessed using an MTT assay. Data are presented as mean ± SD from triplicate wells. Co-treatment with CES1 and sTRAIL mRNA in the presence of CPT-11 resulted in the most significant reduction in cell viability. Statistical analysis was performed using one-way ANOVA followed by Tukey’s post hoc test. *p < 0.05, **p < 0.01, ***p < 0.001, NS = not significant.
